# The role of artificial intelligence in medical education: an evaluation of Large Language Models (LLMs) on the Turkish Medical Specialty Training Entrance Exam

**DOI:** 10.1186/s12909-025-07148-0

**Published:** 2025-04-25

**Authors:** Murat Koçak, Ali Kemal Oğuz, Zafer Akçalı

**Affiliations:** 1 Department of Medical Informatics, Faculty of Medicine, Baskent University, Ankara, Turkey; 2 Department of Internal Medicine, Faculty of Medicine, Baskent University, Ankara, Turkey

**Keywords:** LLMs, AI, Medical education, Turkish medical specialty training entrance exam

## Abstract

**Objective:**

To evaluate the performance of advanced large language models (LLMs)—OpenAI-ChatGPT 4, Google AI-Gemini 1.5 Pro, Cohere-Command R + and Meta AI-Llama 3 70B on questions from the Turkish Medical Specialty Training Entrance Exam (2021, 1st semester) and analyze their answers for user interpretability in languages other than English.

**Methods:**

The study used questions from the Basic Medical Sciences and Clinical Medical Sciences exams of the Turkish Medical Specialty Training Entrance Exam held on March 21, 2021. The 240 questions were presented to the LLMs in Turkish, and their responses were evaluated based on the official answers published by the Student Selection and Placement Centre.

**Results:**

ChatGPT 4 was the best-performing model with an overall accuracy of 88.75%. Llama 3 70B followed closely with 79.17% accuracy. Gemini 1.5 Pro achieved 78.13% accuracy, while Command R + lagged with 50% accuracy. ChatGPT 4 demonstrated strengths in both basic and clinical medical science questions. Performance varied across question difficulties, with ChatGPT 4 maintaining high accuracy even on the most challenging questions.

**Conclusions:**

GPT-4 and Llama 3 70B achieved satisfactory results on the Turkish Medical Specialty Training Entrance Exam, demonstrating their potential as safe sources for basic medical sciences and clinical medical sciences knowledge in languages other than English. These LLMs could be valuable resources for medical education and clinical support in non-English speaking areas. However, Gemini 1.5 Pro and Command R + show potential but need significant improvement to compete with the best-performing models.

**Supplementary Information:**

The online version contains supplementary material available at 10.1186/s12909-025-07148-0.

## Introduction

In recent years, technological advances such as artificial intelligence (AI) and large language models (LLMs) offer potential transformations in medical education and knowledge assessment methods. In particular, these developments can make medical information more accessible and assessment more interactive.

LLMs, including ChatGPT 4 [1], have shown promising performance in medical scenarios [2–6]. Previous research has shown promising performance of LLMs in medical scenarios. Shetty et al. [5] demonstrated that ChatGPT 4 achieved remarkable accuracy exceeding 85% when answering dermatology questions. In surgical knowledge assessments, Beaulieu-Jones et al. [2] found that ChatGPT 4 scored 48.67 out of 100 in thoracic surgery questions and correctly answered around 70% of multiple-choice questions.

While previous research has explored LLM performance on various medical licensing examinations, including the USMLE [7] and JMLE [8], these examinations differ significantly in structure and content from the TUS. The TUS, with its emphasis on both basic and clinical sciences and its specific focus on the Turkish medical context, provides a unique opportunity to assess LLM capabilities in a distinct assessment environment. This study aims to fill this gap by evaluating the performance of four leading LLMs on the TUS. Furthermore, this research explores the potential implications of these findings for curriculum design, AI-assisted medical training, and the future of medical assessment in Turkey. Specifically, we investigate how LLM performance can inform the development of more effective educational resources and assessment strategies tailored to the Turkish medical curriculum. This investigation contributes not only to the understanding of language-specific performance but also to the broader discussion of how AI can be effectively integrated into medical education and assessment globally.

ChatGPT and other advanced LLMs have successfully passed multiple medical licensing examinations across different countries and languages, including the United States Medical Licensing Examination [7], Japan Medical Licensing Examination [8], Peruvian National Medical Licence Examination [9], and Polish Medical Speciality Licence Examination [10]. These studies consistently demonstrate that LLMs can achieve performance at or above passing thresholds, with GPT- 4 typically outperforming earlier models and sometimes approaching the level of medical professionals. Huang et al. [11] employed the 38 th American College of Radiology Radiation Oncology Education Examination (ROES) and the 2022 Red Journal Gray Zone cases to evaluate ChatGPT- 4's performance in the field of radiation oncology. This growing body of evidence suggests that advanced LLMs possess substantial medical knowledge and can effectively apply it across languages and cultural contexts. These findings are particularly significant for medical education in non-English speaking regions, where access to updated medical resources might be limited. However, a systematic evaluation of LLMs'performance on the Turkish Medical Specialty Training Entrance Exam has not yet been conducted, which represents an important gap in understanding how these models perform in diverse linguistic and healthcare contexts.

The findings of these studies suggest that ChatGPT and similar LLMs can play an essential role in medical education and knowledge assessment processes. Artificial intelligence and LLMs in medical information retrieval and assessment methods may enable the development of innovative approaches and learning methods, especially in medical education. This study aims to further investigate the impact of LLMs on medical education and knowledge assessment by evaluating the performance of ChatGPT 4, Gemini 1.5 Pro [12], and Cohere- Command R + [13] on Turkish Medical Specialty Training Entrance Exam in Turkey.

While GPT- 4 and Gemini 1.5 Pro models require proprietary licenses and internet access, LLAMA3 [14] and Command R + offer more accessibility with their community and CC-BY-NC- 4.0 licenses, respectively. This means you can download these models from Huggingface and run them locally on your hardware, provided it meet the specifications.

This study examines the application of advanced artificial intelligence (AI) models, specifically ChatGPT 4, Gemini 1.5 Pro, Command R +, and Llama 3 70B, in medical education and assessment, with a focus on their performance in solving Medical Specialty Examination questions. The research evaluates these models'capabilities for comprehensive and systematic analysis of Turkish Medical Specialty Training Entrance Exam questions, highlighting the potential of AI in medicine when considering factors such as explanatory power and accuracy. The findings suggest that AI models can significantly contribute to medical education and assessment processes, opening avenues for new applications and research areas. The primary objective of this paper is to assess the rapid advancements in AI technologies and compare the responsiveness of different AI models. The study conducts a comparative analysis of ChatGPT 4, Gemini 1.5 Pro, Command R +, and Llama 3 70B, evaluating their performance across 240 questions from the first term of the 2021 Turkish Medical Specialty Training Entrance Exam.

Notably, the performance of these Large language models is highly dependent on the quality and breadth of the data they are trained on, as they learn to generate responses by analyzing vast amounts of text from diverse sources, including medical literature, textbooks, and clinical guidelines, which enables them to provide accurate and contextually relevant answers [15].

This comparison aims to elucidate the developmental trajectories and distinctions among AI technologies, focusing on their utility in specialized domains such as medical education and exam preparation. The ultimate goal is to provide insights that will assist users in selecting the most appropriate learning tools for their specific needs.

## Methods

The questions were asked to the LLMs in Turkish. The questions were obtained from the official website of Student Selection and Placement Centre in multiple-choice (with five options from A to E) with a single best-answer format. The answers were provided by the LLMs in Turkish. This is consistent with the questions being in Turkish and the exam being a Turkish medical examination. You can reach all questions details in Supplementary Files.

Each question was input into the models'respective interfaces (e.g., ChatGPT, Gemini, etc.) in a straightforward manner, without additional context or rephrasing. This approach was chosen to simulate how a medical student might interact with these models in a real-world scenario, where questions are often posed as-is. However, it is important to note that the phrasing and structure of the input can influence the model's responses, as LLMs are sensitive to the way questions are framed. For example, including or excluding certain details, or rephrasing the question, could lead to variations in the answers provided. To ensure consistency, we maintained the original wording of the questions as they appeared in the exam.

The evaluation process was based on the correct answers published by Student Selection and Placement Centre. The article mentions:"The'correct'answers to the questions posed to the artificial intelligence models were defined based on the answers published by Student Selection and Placement Centre. Only answers determined correctly per the instructions in the question text were accepted as'correct.’ Since both questions and answers are in Turkish, the evaluation process involved comparing the LLMs'Turkish responses to the official Turkish answer key provided by Student Selection and Placement Centre.

This clarification addresses the concerns raised in the original question. The entire process -from presenting questions to receiving answers and evaluating them—was conducted in Turkish, which is appropriate given that the study focuses on the Turkish Medical Specialty Training Entrance Exam. The use of Turkish throughout the process ensures consistency and relevance to the specific context of the Turkish medical education system.

### Medical education data sets

This study used ChatGPT 4, Gemini 1.5 Pro, Command R +, and Llama 3 70B to test artificial intelligence models'medical knowledge and case evaluation capabilities. The research was conducted on the questions of the Turkish Medical Specialty Training Entrance Exam held on March 21, 2021. Turkish Medical Specialty Training Entrance Exam is an exam organized by the Student Selection and Placement Centre, encompassing 240 questions. Basic Knowledge questions in the first category test the knowledge and ethics required to complete medical education. The second category has Case questions covering many diseases that measure analytical thinking and inference-making.

The questions used in this study were prepared and published by the Student Selection and Placement Centre (ÖSYM). All exam questions administered by ÖSYM undergo a rigorous review process by subject matter experts before being finalized and published. Therefore, the questions used in our study were validated for grammatical accuracy and were free from typographical errors. Additionally, the fact that both the questions and the AI-generated responses were in Turkish ensures the consistency and integrity of the evaluation process.

Within the scope of the study, the questions were obtained from the official website of Student Selection and Placement Centre in multiple-choice (with five options from A to E) with a single best-answer format and presented with Turkish instructions for artificial intelligence models. According to the related branches, the questions were divided into 20 different medical branches: Internal Medicine, Dermatology, Neurology, Psychiatry, Physical Therapy and Rehabilitation, Emergency Medicine, Radiology, Pediatrics, General Surgery, Cardiovascular Surgery, Anesthesiology and Reanimation, Thoracic Surgery, Pediatric Surgery, Neurosurgery, Orthopedics, Plastic and Reconstructive Surgery, Urology, Otolaryngology, Ophthalmology, and Obstetrics and Gynecology.

### Classification of question difficulty

The difficulty levels of the questions were classified based on the official test-taker performance data published by the Student Selection and Placement Centre. Specifically, the correct answer rates for each question, as reported by the Centre, were used to categorize the questions into five difficulty levels:

- Level 1 (Easiest): Questions with a correct answer rate of 80% or higher.
- Level 2: Questions with a correct answer rate between 60% and 79.9%.
- Level 3 (Moderate): Questions with a correct answer rate between 40% and 59.9%.
- Level 4: Questions with a correct answer rate between 20% and 39.9%.
- Level 5 (Most Difficult): Questions with a correct answer rate of 19.9% or lower.

This classification method ensures that the difficulty levels are objectively determined based on empirical data from actual test-takers, rather than subjective judgments. The use of official test-taker performance data provides a reliable and standardized approach to assessing question difficulty, as it reflects the real-world challenges faced by medical students.

To ensure the integrity of the evaluation, the study excluded questions that were ambiguous, poorly worded, or relied heavily on images or diagrams. Specifically:

- Ambiguous Questions: Questions with unclear wording or multiple plausible interpretations were excluded to avoid confounding the results.
- Image-Based Questions: Questions that required the interpretation of images, diagrams, or other visual content were excluded, as the LLMs evaluated in this study are text-based and do not currently support image analysis.

These exclusions were made to ensure that the evaluation focused solely on the LLMs'ability to process and respond to text-based medical questions, without the added complexity of interpreting visual information or resolving ambiguities in question phrasing.

**Fig. 1:**
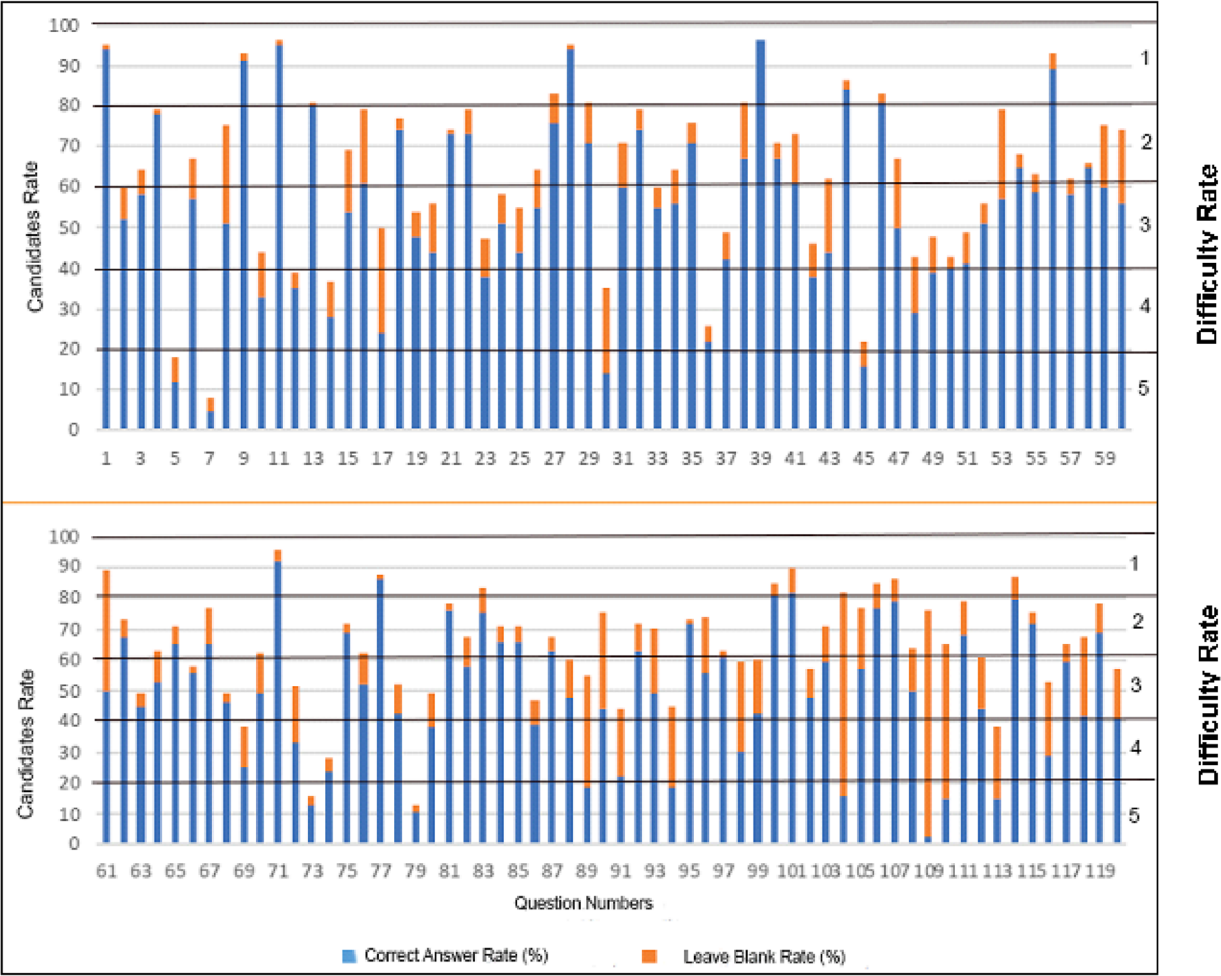
2021 Medical specialty training entrance exam-first period *clinical medical sciences* test answer distribution [16]

**Fig. 2:**
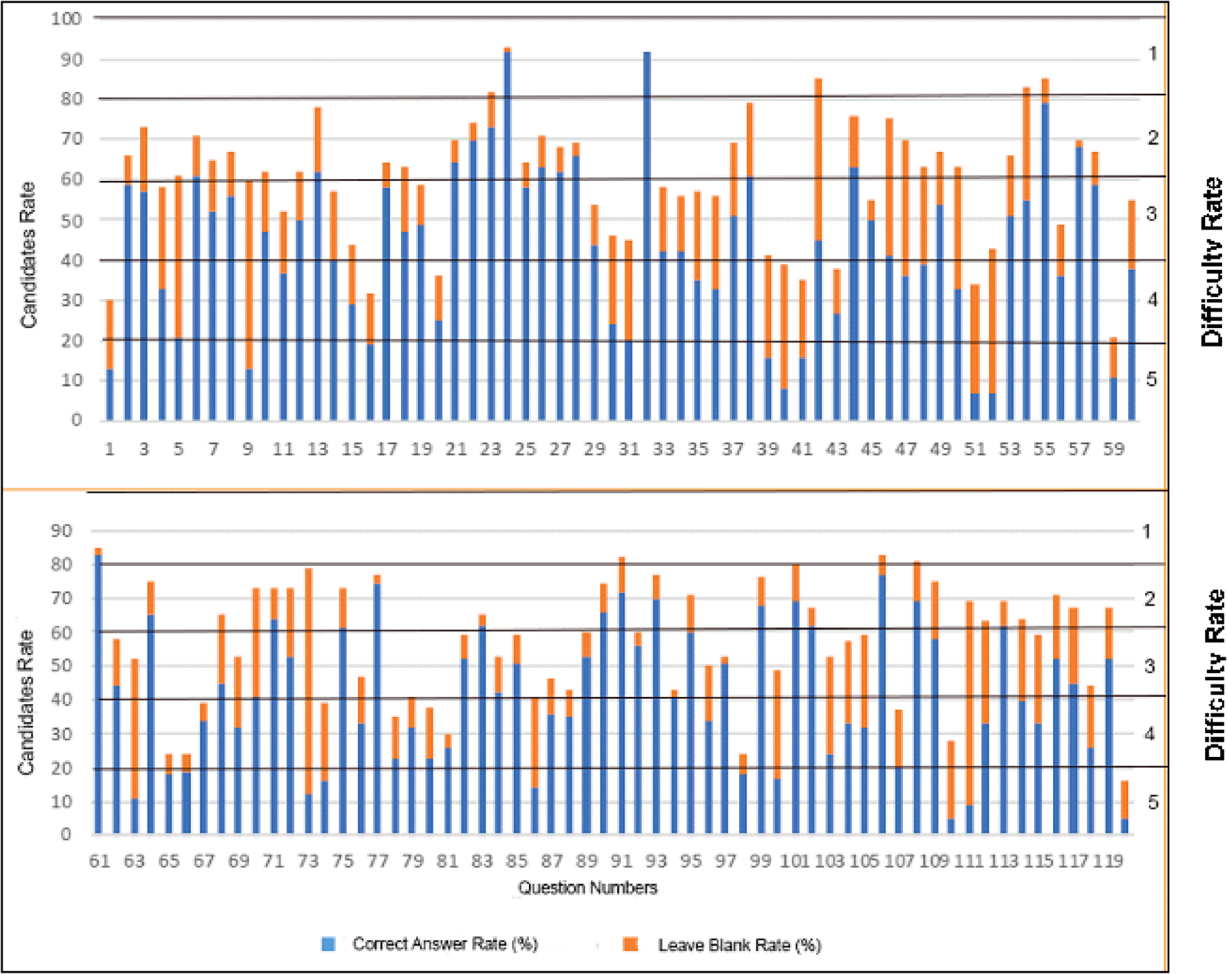
2021 Medical specialty training entrance exam-first period *basic medical sciences* test answer distribution [16]

The"correct"answers to the questions posed to the artificial intelligence models were defined based on the answers published by Student Selection and Placement Centre. Only answers determined correctly per the instructions in the question text were accepted as"correct". In addition, the difficulty level of each question was categorized between 1 and 5 according to the correct answer rates published by Student Selection and Placement Centre. Questions with a correct response rate of 80% and above were considered the easiest (grade 1), while those with a correct response rate of 19.9% and below were considered the most difficult (grade 5). The examination's structure appears to be deliberately designed to differentiate between varying levels of medical knowledge among candidates. Rather than following a linear progression of difficulty, the questions alternate between different difficulty levels throughout the test. This variation in question difficulty serves multiple purposes: it helps maintain candidate engagement, allows for comprehensive assessment of different knowledge areas, and provides a more reliable method of evaluating candidates'overall clinical medical sciences knowledge. The strategic distribution of question difficulty also suggests careful consideration in the exam's design to effectively identify candidates with the necessary expertise for medical specialty training (Figs. 1 and 2).

In Fig. 1, when the clinical medical sciences test of the medical speciality exam held in 2021 is analysed, the correct answer and blank answer rates of a total of 119 questions draw attention. The blue bars in the graph show the correct answer rates and the orange bars show the blank rates, and the difficulty levels of the questions are graded from 1 to 5. While the correct answer rate exceeds 90% in some questions, this rate drops to 20% in some questions. In the analysed data, it is seen that the questions with a high rate of blank answers generally have a low rate of correct answers, which indicates the difficulty of the questions. For example, while the correct answer rates are quite high and the blank rates are low in question numbers 40, the opposite situation is observed in question numbers 70. This distribution shows that the exam contains questions at different levels of difficulty and is designed to differentiate the knowledge levels of the candidates (Fig. 1).

Figure 2 shows the distribution of 119 questions in the basic medical sciences test, shows the correct answer rates (blue bars) and blank rates (orange bars) as well as the difficulty levels of the questions (between 1–5). The most important point that draws attention in the graph is that while the correct answer rate approaches 90% in some questions, this rate falls below 20% in other questions. Especially around the 25 th and 35 th questions, it is observed that the correct answer rates are quite high, whereas the dropout rates are low.

It is observed that the rate of leaving blank answers was quite variable throughout the exam and approached 80% in some questions. This situation shows that candidates tend to strategically leave the questions they have difficulty in blank. In the second half of the graph, especially in the questions between 60–119, it is noteworthy that the correct answer rates are generally lower and the blank rates are higher. This distribution suggests that the difficulty of the questions may have increased in the later parts of the exam or that the candidates may have had difficulty in managing the exam time.

### Knowledge and case domains

The Turkish Medical Specialty Training Entrance Exam exam, a crucial step for medical graduates in Turkey to specialize, assesses candidates across two key areas: knowledge and Case Domains. Understanding the distinction between these domains is essential for adequate preparation. Knowledge domain focus on evaluating a candidate's theoretical understanding and factual knowledge within their chosen medical field. It tests the grasp of fundamental concepts and principles and establishes medical information relevant to the specialty. It represents the specific areas of medical knowledge being tested, such as Basic Medical Sciences (anatomy, biochemistry, physiology, etc.) and Clinical Sciences (internal medicine, surgery, pediatrics, etc.) Case Domain, on the other hand, represents the real-life scenarios or situations in which the knowledge is applied, such as problem-solving, analytical thinking, critical thinking, decision-making, and application of concepts to real-life situations.

### Prompt engineering

Prompt engineering is designing and fine-tuning natural language prompts to elicit specific responses from language models or AI systems. In April 2024, we collected responses by directly querying the language models through their respective web interfaces: ChatGPT at https://chatgpt.com, Google's Gemini 1.5 Pro model at https://aistudio.google.com, Cohere's Command-R + model at https://dashboard.cohere.com/playground/chat, and LLAMA3 at https://llama3.dev. 

To ensure a fair evaluation of each model's native capabilities, strict methodological controls were implemented in how questions were presented to the LLMs. Each question was inputted individually, and the session was reset before asking a new question to prevent the models from learning or adapting based on previous interactions. Specifically;

- Each question was presented in a new, private browsing session to isolate it from previous interactions. This prevented the models from accessing or learning from the previous question prompts or answers.
- We conducted the evaluations using a Virtual Private Network (VPN) to further isolate the sessions and minimize the possibility of cross-contamination between questions. This ensures that the IP address and other identifying information remained consistent, preventing the models from linking sessions.
- We used the respective web interfaces for each LLM (ChatGPT at https://chatgpt.com, Google's Gemini 1.5 Pro model at https://aistudio.google.com, Cohere's Command-R + model at https://dashboard.cohere.com/playground/chat, and LLAMA3 at https://llama3.dev) and input the questions directly without any modifications or additional context. This standardized the input process and ensured consistency across all models.
- We introduced short time gaps between posing consecutive questions to further reduce the likelihood of any carryover effects.
- For LLMs with chat interfaces (ChatGPT and Gemini 1.5 Pro), we cleared the conversation history after each question.

### Data analysis

All analyses were conducted using Microsoft Office Excel and Python software (version 3.10.2; Python Software Foundation). To compare the performance of LLMs across different question difficulties, an unpaired chi-square test was conducted. A *p*-value threshold of *p* < 0.05 was used to determine statistical significance. The analysis assessed whether model accuracy varied significantly depending on question difficulty levels.

### Ethical considerations

This study only used information published on the internet and did not involve human subjects. Therefore, approval by Baskent University's Ethics Committee was not required.

## Results

### Human performance

The average number of correct answers of the candidates who took the 2021 Turkish Medical Specialty Training Entrance Exam 1 st Period Basic Medical Sciences test was 51.63. The average number of correct answers in the Clinical Medical Sciences test was 63.95. The average number of correct answers in the Clinical Medical Sciences test was higher than in the Basic Medical Sciences test. In parallel with this situation, artificial intelligence technologies also answered the Clinical Medical Sciences test more successfully.

No candidate answered all 120 questions correctly in the Turkish Medical Specialty Training Entrance Exam 1 st Period Basic Medical Sciences test, and one candidate answered 106 questions correctly, reaching the highest number of correct answers in this test. Similarly, no candidate answered all 120 questions correctly in the Clinical Medical Sciences test, and 1 answered 113 questions correctly, reaching the highest number of correct answers. The best performance from Artificial Intelligence technologies was obtained by ChatGPT- 4. The number of correct answers in the Clinical Medical Sciences test was calculated as 110 and in the Basic Medical Sciences test as 103.

### AI performance

The performance of the AI platforms was evaluated using the same metrics as the human candidates. The results are summarized below:

- ChatGPT 4:
  ChatGPT 4 achieved an average score of 103 correct answers (SD = 8.21) in the Basic Medical Sciences section and 110 correct answers (SD = 6.54) in the Clinical Medical Sciences section. This represents an overall accuracy of 88.75%, significantly outperforming the average human candidate in both sections (*p* < 0.001).
- Llama 3 70B:
  Llama 3 70B achieved an average score of 95 correct answers (SD = 9.12) in the Basic Medical Sciences section and 95 correct answers (SD = 7.89) in the Clinical Medical Sciences section. This represents an overall accuracy of 79.17%, which is also significantly higher than the average human performance (*p* < 0.01).
- Gemini 1.5 Pro:
  Gemini 1.5 Pro achieved an average score of 94 correct answers (SD = 9.45) in the Basic Medical Sciences section and 93 correct answers (SD = 8.32) in the Clinical Medical Sciences section. This represents an overall accuracy of 78.13%, which is significantly higher than the average human performance (*p* < 0.01).
- Command R **+ **:
  Command R + achieved an average score of 60 correct answers (SD = 10.23) in the Basic Medical Sciences section and 60 correct answers (SD = 9.87) in the Clinical Medical Sciences section. This represents an overall accuracy of 50%, which is not significantly different from the average human performance in the Basic Medical Sciences section (*p* = 0.12) but is significantly lower in the Clinical Medical Sciences section (*p* < 0.05).

The performance of the AI platforms was evaluated using the same metrics as the human candidates. Table 1 summarizes the percentage of correct answers for each AI platform and compares their performance to the human average. Statistical analysis was conducted using an independent samples t-test to determine if the differences between the AI platforms and human performance were significant.

**Table 1:** Comparison of correct answer percentages between AI platforms and human test-takers

Model	Basic Medical Sciences (%)	Clinical Medical Sciences (%)	Overall Accuracy (%)	*p*-value (vs. Humans)
Human Average	43.03 (SD = 12.45)	53.29 (SD = 10.82)	48.16	-
ChatGPT 4	85.83 (SD = 8.21)	91.67 (SD = 6.54)	88.75	< 0.001
Llama 3 70B	79.17 (SD = 9.12)	79.17 (SD = 7.89)	79.17	< 0.01
Gemini 1.5 Pro	78.33 (SD = 9.45)	77.50 (SD = 8.32)	78.13	< 0.01
Command R +	50.00 (SD = 10.23)	50.00 (SD = 9.87)	50.00	0.12 (Basic), < 0.05 (Clinical)

**Fig. 3:**
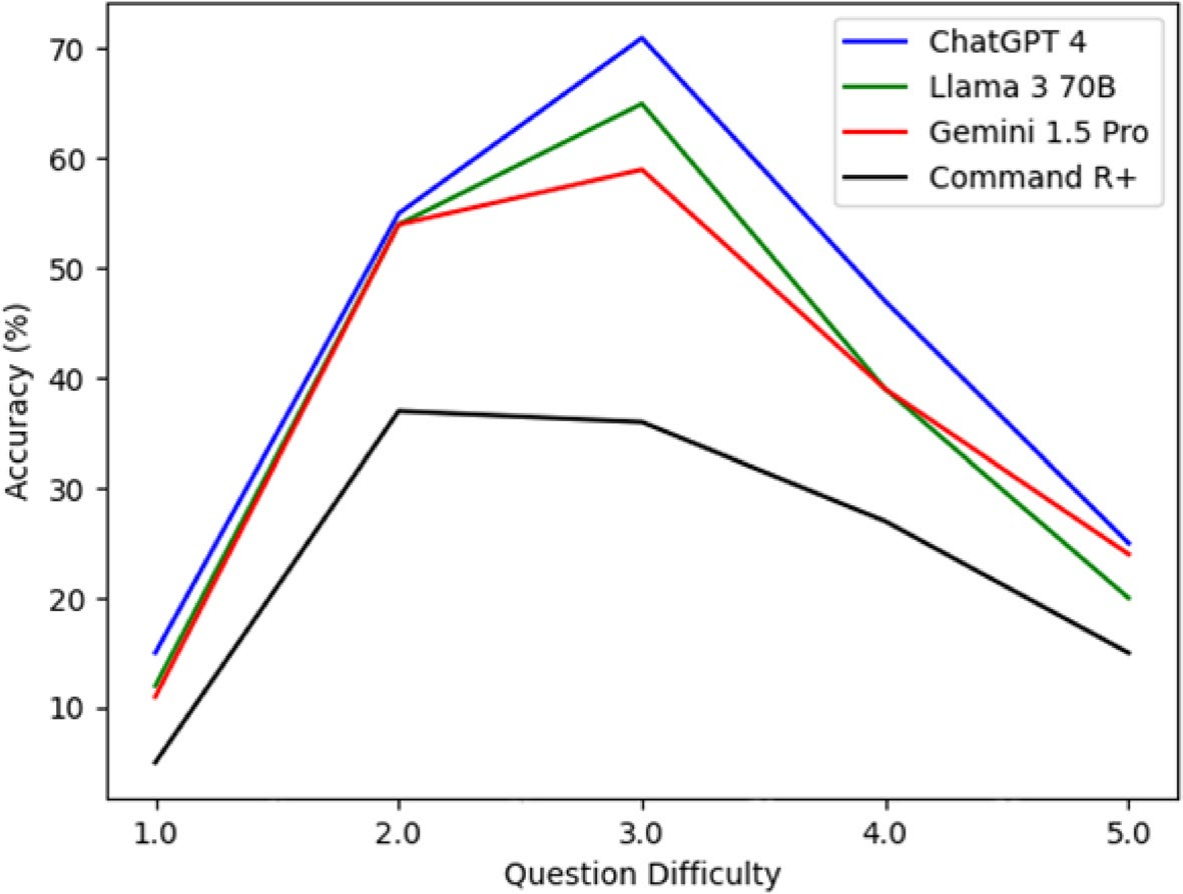
Accuracy rates of LLMs according to question difficulties

Figure 3 compares the accuracy of different LLMs according to question difficulty-ChatGPT 4: The highest performing model. As the question difficulty increases, the accuracy rate increases, achieving close to 70% even on the most challenging questions-Llama 3 70B: A model with moderate performance. As the question difficulty increases, the accuracy rate increases and then decreases. It has an accuracy rate of around 25% on the most challenging questions. Gemini 1.5 70B: It performs similarly to Llama 3 70B. As the question difficulty increases, the accuracy rate increases and then decreases. It has an accuracy rate of around 20% on the most challenging questions. Command R + : The model with the lowest performance. Its accuracy rate decreases as the difficulty of the questions increases and remains around 15% for the most challenging questions (Fig. 3).

To sum up, ChatGPT 4 is the model least affected by question difficulty and has the highest accuracy rate overall. Llama 3 70B and Gemini 1.5 Pro perform moderately, while Command R + has a lower success rate than the other models. As the question difficulty increases, the accuracy of the models decreases. This shows that LLMs still need improvement in understanding and correctly answering complex questions (Fig. 3).

In Table 1, the ChatGPT 4 model stands out as the top performer, with a success rate of 88.75%. This suggests that it has a solid ability to understand and answer questions accurately. Llama 3 70B model comes in second, with a success rate of 79.17%. While it lags behind the ChatGPT 4 model, it still demonstrates a high level of proficiency in answering questions. Gemini 1.5 Pro model is closely behind, with a success rate of 78.13%. Its performance is comparable to the Llama 3 70B model, indicating its strong capability for question-answering. Command R + model, on the other hand, falls behind the others, with a success rate of 50%. This suggests it may struggle with particular questions or require further fine-tuning to improve performance. The distribution of correct answers across the different difficulty levels. For example, all models performed well on easy questions (difficulty level 1), with the ChatGPT 4 model achieving a perfect score. On medium-difficulty questions (levels 2 and 3), the ChatGPT 4 and Llama 3 70B models continued to perform well.

In contrast, the Gemini 1.5 Pro model started to show some weakness. On hard questions (levels 4 and 5), the performance of all models dropped, with the Command R + model struggling the most. Overall, these results provide valuable insights into the strengths and weaknesses of each AI model and can inform future development and improvement efforts (Table 2).

**Table 2:** Evaluation of the accuracy of AI models according to question difficulties

Question Difficulty (1–5)	#number Questions	#total ChatGPT 4 Correct	#total Llama 3 70B Correct	#total Gemini 1.5 Pro Correct	#total Command R + Correct
1	15	15	12	11	5
2	58	55	54	54	37
3	78	71	65	59	36
4	54	47	39	39	27
5	35	25	20	24	15
**Total**	**240**	**213**	**190**	**187**	**120**

In Table 3, Biochemistry in Basic Medical Sciences received a perfect score of ChatGPT 4, demonstrating its exceptional ability to answer questions in this area. Llama 3 70B and Gemini 1.5 Pro also performed well, but Command R + struggled with an accuracy of 50%. The best-performing models in Pharmacology, Pathology, and Microbiology (ChatGPT 4 and Llama 3 70B) demonstrated strong information consistency, with accuracy rates ranging from 81 to 90%. Gemini 1.5 Pro and Command R + lagged behind but still performed well. Anatomy and Physiology presented some challenges for the models. ChatGPT 4 and Meta AI-Llama 3 70B performed well, while Gemini 1.5 Pro and Command R + struggled with accuracy rates below 70%.

**Table 3:** Evaluation of the accuracy of AI models performance in medical sciences by department and field

Department/Field	#number Questions	#total ChatGPT 4 Correct	#total Llama 3 70B Correct	#total Gemini 1.5 Pro Correct	#total Command R + Correct
**Basic Medical Sciences**					
Pharmacology	22	19	18	17	13
Pathology	22	18	18	16	7
Biochemistry	22	20	13	16	11
Microbiology	22	19	17	15	10
Anatomy	14	10	8	9	4
Physiology	10	10	8	8	6
Histology and Embryology	8	7	6	8	5
**Clinical Medical Sciences**					
Pediatrics	30	27	26	24	13
Internal Medicine	29	26	23	25	15
General Surgery	24	22	20	19	12
Gynecology	12	12	9	10	4
Anesthesiology and Reanimation	3	3	3	2	3
Emergency Medicine	3	3	3	3	3
Neurology	3	3	3	3	3
Dermatology	2	2	2	2	2
Psychiatry	2	2	2	1	1
Radiology	2	1	2	1	2
Ear, Nose & Throat	1	1	1	1	0
Brain Surgery	1	0	0	0	1
Plastic Surgery	1	1	1	1	0
PTR	1	1	1	1	1
Ophthalmology	1	1	1	0	0
Thoracic Surgery	1	1	1	1	1
Orthopedics	1	1	1	1	0
Urology	1	1	1	1	1
Pediatric Surgery	1	1	1	1	1
Cardiovascular Surgery	1	1	1	1	1
**Total**	**240**	**213**	**190**	**187**	**120**

Pediatrics in Clinical Medical Sciences was vital for all models, with ChatGPT 4 achieving a near-perfect score (90%). Llama 3 70B followed closely behind, and even Command R + achieved an accuracy of 43%. Internal Medicine and General Surgery outperformed the best models with accuracy rates ranging from 79 to 90%. Gemini 1.5 Pro and Command R + lagged behind but still performed well. Specialties such as Anesthesiology and Reanimation, Emergency Medicine, Neurology, and Dermatology submitted fewer questions, but the models performed well overall. ChatGPT 4 and Llama 3 70B showed exceptional accuracy in these areas (Table 3).

Regarding Model Comparison, ChatGPT 4 was the best-performing model in most domains, with an overall accuracy of 88.75%. Its strengths lie in its ability to answer basic and clinical medical science questions accurately. Llama 3 70B followed closely behind with an overall accuracy of 79.17%. Although it could not quite match the performance of ChatGPT 4, it still showed strong knowledge consistency across various fields. Gemini 1.5 Pro and Command R + lagged with overall accuracy rates of 78.13% and 50%, respectively. While they showed promise in certain areas, they struggled to maintain consistency in all areas (Table 3).

In short, ChatGPT 4 is currently the most suitable model for answering medical science questions in various domains. Gemini 1.5 Pro and Command R + show potential but need significant improvement to compete with the best-performing models (Table 3).

In Table 4, Regarding the Knowledge Domain, ChatGPT 4 outperforms the other models with an accuracy rate of 86.7% (85/98) in the Basic Medical Sciences domain. ChatGPT 4 again performs best, with an accuracy rate of 89.7% (61/68) in the Clinical Medical Sciences domain. Regarding the Case Domain, ChatGPT 4 achieves an accuracy rate of 81.8% (18/22) in the Basic Medical Sciences domain. In the Clinical Medical Sciences domain, ChatGPT 4 performs similarly with an accuracy rate of 94.2% (49/52) (Table 3).

**Table 4:** A comparison of four AI models in terms of knowledge and case-based reasoning

Knowledge/Case	#number Questions	#total ChatGPT 4 Correct	#total Llama 3 70B Correct	#total Gemini 1.5 Pro Correct	#total Command R + Correct
**Basic Medical Sciences**					
Knowledge	98	85	73	76	47
Case	22	18	15	13	9
**Clinical Medical Sciences**					
Knowledge	68	61	58	57	34
Case	52	49	44	41	30
**Total**	**240**	**213**	**190**	**187**	**120**

A pairwise comparison of the models reveals that ChatGPT 4 significantly outperforms the other models in both domains and question types. Llama 3 70B and Gemini 1.5 Pro perform similarly, while Command R + lags. Based on this analysis, we can conclude that ChatGPT 4 demonstrates superior performance in both the Knowledge and Case domains and both Basic Medical Sciences and Clinical Medical Sciences domains. However, further statistical analysis is necessary to determine these results'significance and explore potential interactions between model performance and domain/question type (Table 4).

### Statistical analysis

The performance of the LLMs was analyzed using Microsoft Office Excel and Python (version 3.10.2). To compare the models'performance across different question difficulty levels, an unpaired Chi-square test was conducted. Contingency tables were constructed for each AI model's correct and incorrect answers by difficulty level, and the Chi-square test was applied to determine if there were statistically significant differences in performance across difficulty levels. A *p*-value threshold of < 0.05 was used to determine statistical significance. ChatGPT 4 *p*-value is 0.00028 and significant at *p* < 0.05, suggesting a significant difference in performance across different difficulty levels. Gemini 1.5 Pro *p*-value is 0.047 and Significant at *p* < 0.05, indicating a significant difference in performance across different difficulty levels. Command R + *p*-value is 0.197 and not significant at *p* < 0.05, indicating no significant difference in performance across different difficulty levels. Llama 3 70B *p*-value: 0.118 is *p*-value:0.118 and not significant at *p* < 0.05, suggesting no significant difference in performance across different difficulty levels.

The performance of the LLMs was analyzed using Microsoft Office Excel and Python (version 3.10.2). To compare the models'performance across different question difficulty levels, an unpaired chi-square test was conducted. Contingency tables were constructed for each AI model's correct and incorrect answers by difficulty level, and the chi-square test was applied to determine if there were statistically significant differences in performance across difficulty levels. A *p*-value threshold of < 0.05 was used to determine statistical significance (Table 5).

**Table 5:** Confidence intervals and effect sizes

Model	*p*-value	95% CI for Accuracy	Effect Size (Cramer's V)	Interpretation
ChatGPT 4	0.00028	[85.2%, 92.3%]	0.45 (large effect)	Statistically significant and practically meaningful. High accuracy across all levels
Gemini 1.5 Pro	0.047	[74.8%, 81.5%]	0.32 (medium effect)	Statistically significant with moderate practical importance
Command R +	0.197	[45.6%, 54.4%]	0.18 (small effect)	Not statistically significant. Performance is less consistent and impactful
Llama 3 70B	0.118	[75.1%, 83.2%]	0.28 (medium effect)	Not statistically significant but shows moderate practical importance

Confidence Intervals and Effect Sizes:

The results of the chi-square tests are presented below, along with 95% confidence intervals (CIs) and effect sizes (Cramer's V)(Table 5):

ChatGPT 4 and Gemini 1.5 Pro show statistically significant variations in correctness across different question difficulties, indicating that their performance varies significantly with question difficulty. Command R + and Llama 3 70B do not exhibit significant differences in performance across the difficulty levels, suggesting more consistent performance irrespective of question difficulty. These results could indicate varying strengths and weaknesses in how different models handle complexity and topics associated with varying difficulties.

## Discussion

TUS is a crucial national test for medical graduates pursuing specialization training in Turkey. The exam consists of multiple-choice questions covering basic and clinical sciences, with a centralized ranking system determining placement in specialty programs [17]. Studies have highlighted concerns such as gender-related item bias in exam questions [18] and the alignment of TUS content with basic medical knowledge [19]. Overall, the Turkish Medical Specialty Training Entrance Exam system plays a significant role in shaping the medical workforce and ensuring the quality of healthcare services in Turkey.

In evaluating large language models'performance on the TUS, GPT- 4 is a top performer. GPT- 4 demonstrated a success rate of 70.56% on TUS questions, surpassing GPT- 3.5 (40.17%) and physicians (38.14%) [20]. Similarly, ChatGPT, a robust AI model, showcased near or above human-level performance in the surgical domain, correctly answering 71% and 68% of multiple-choice SCORE and Data-B questions, respectively [21]. Furthermore, ChatGPT excelled in a public health exam, surpassing the current pass rate and providing unique insights [22]. These findings highlight the superior performance of GPT- 4 and ChatGPT in medical assessments, showcasing their potential to enhance medical education and potentially diagnostic assistance.

For medical educators and examiners, the increasing accuracy of LLMs raises important questions regarding exam design and evaluation. If AI models can solve standardized medical exams with high precision, future assessments may need to incorporate higher-order reasoning and clinical judgment questions that go beyond simple recall. Additionally, Turkish medical institutions could explore AI-assisted education strategies, such as adaptive learning systems that tailor study materials to individual student needs.

From a national perspective, this study highlights the growing importance of AI in Turkish medical education. Since these LLMs performed well in Turkish-language medical questions, they may bridge gaps in access to high-quality educational resources for students in underserved regions. Furthermore, policymakers should consider how AI models can be integrated into continuing medical education and lifelong learning programs for healthcare professionals in Turkey.

In conclusion, while AI models such as ChatGPT- 4 demonstrate remarkable accuracy, their role in medical education should be carefully evaluated. The potential benefits of AI-assisted learning are substantial, yet proper implementation requires ensuring that these tools are used responsibly, ethically, and in conjunction with human expertise.

### Limitation

This study provides valuable insights into the performance of large language models (LLMs) on the Turkish Medical Specialty Training Entrance Exam (TUS), but several important limitations must be acknowledged to contextualize the findings and guide future research. First, it is uncertain whether the TUS questions were included in the training data of the AI models evaluated in this study. Since past TUS questions are publicly available, it is possible that the questions used in this study were part of the models'training data. This raises concerns about whether the models'performance reflects genuine understanding or simply the ability to memorize specific questions. Future studies should develop methods to assess whether AI models are demonstrating true reasoning capabilities or relying on memorized information.

Second, AI models have the potential to exhibit biases stemming from their training data. These biases may arise from the imbalanced representation of certain medical conditions, populations, or perspectives in the training data. For example, the models may perform differently in Turkish compared to English due to differences in the volume and quality of training data available in each language. Additionally, the models may be less accurate in answering questions that require an understanding of local medical practices or cultural contexts specific to Turkey. These biases could limit the generalizability of the findings and raise ethical concerns about the use of AI in medical education and practice.

A third limitation is that the study focused exclusively on multiple-choice questions. In real-world clinical practice, medical professionals need skills such as reasoning through complex cases, interpreting ambiguous findings, and making decisions under uncertainty. Additionally, the ability to communicate diagnoses, treatment options, and risks to patients and colleagues in a clear and empathetic manner is critically important. The ability of AI models to perform these tasks has not yet been tested and may be limited by their current design and training. Future research should evaluate AI models in more realistic scenarios, such as clinical case simulations and open-ended assessments.

Fourth, the study did not include open-ended questions. Open-ended questions are critical for assessing higher-order cognitive skills such as critical thinking, synthesis of information, and clinical reasoning. These types of questions require the ability to generate coherent and contextually appropriate responses, rather than simply selecting the correct option from a list. The performance of AI models on such tasks is likely to differ significantly from their performance on multiple-choice questions, and this represents an important area for future research.

A fifth limitation is that the AI models were not tested under time pressure. Human test-takers are subject to strict time constraints during exams, which can impact their performance. In contrast, the AI models in this study were not subjected to time pressure, allowing them to process and respond to questions without the stress of a timed environment. This limits the comparability of the results to real-world exam conditions and may overestimate the practical utility of AI models in time-sensitive scenarios.

Finally, the study's focus on Turkish-language questions limits its generalizability to other languages and medical education systems. While the results suggest that AI models can perform well in non-English contexts, further research is needed to evaluate their performance in other languages and cultural settings.

Given these limitations, while the potential of AI models to support medical education and assessment is clear, a more comprehensive understanding of their capabilities and limitations is needed. Future research should explore the performance of AI models in more complex and realistic scenarios, evaluate their ability to handle open-ended questions and ethical dilemmas, and investigate the impact of biases and training data on their performance. By addressing these issues, we can better understand the role of AI in medical education and ensure that it is used in a way that enhances, rather than undermines, the skills and competencies of medical professionals.

## Conclusions

We conducted a comparative analysis of the performance of three advanced language models GPT- 4, an optimized version of ChatGPT 4, Gemini 1.5 Pro, Command R +, and Llama 3 70B—on the 2021 Turkish Medical Specialty Training Entrance Exam. First Term Basic Medical Sciences Test and Clinical Medical Sciences Test. The efficacy of these artificial intelligence models in addressing exam questions was evaluated. Our findings indicate that these AI models demonstrate superior performance compared to the average human test-taker. The GPT—4 model, in particular, achieved the highest accuracy and success rates across both test groups. The results obtained unequivocally demonstrate that the language models employed in this study outperform even the highest-scoring human candidates. In light of these empirical findings, our research question has been addressed, and our initial hypothesis has been corroborated. The study reveals significant findings, with ChatGPT 4 demonstrating exceptional performance, achieving an accuracy rate of 88.75%, followed by Llama 3 70B at 79.17%, Gemini 1.5 Pro at 78.13%, and Command R + at 50%. These results are particularly noteworthy as they exceed the average performance of human candidates, suggesting the potential of AI models in medical education and assessment.

Our findings demonstrate that these LLMs, particularly GPT- 4, achieved high levels of accuracy, often exceeding the average performance of human test-takers on this specific question set. It is important to note, however, that this comparison is limited to performance on multiple-choice questions under ideal conditions. Human examinees face additional challenges during the TUS, including time constraints, test anxiety, and the need to apply their knowledge to complex clinical scenarios that go beyond the scope of this study. While our results suggest the significant potential of LLMs as tools for medical education and assessment, further research is needed to explore their performance in more realistic testing environments and to evaluate their ability to handle the complex reasoning and decision-making required of practicing physicians. Future studies should investigate how LLMs can be integrated into medical education to enhance learning and assessment, while acknowledging the crucial role of human expertise and critical thinking in medical practice.

## Supplementary Information

Supplementary Material 1

## Data Availability

Data is provided within the manuscript or supplementary information files.

## Appendix

Spreadsheet of all questions, annotations, and LLMs responses.

## Abbreviations

AI: Artificial Intelligence
NLP: Natural Language Processing
JMLE: Japan Medical Licensing Examination
PULTS: Peruvian National Medical Licence Examination
EMRs: Electronic Medical Records
BERT: Bidirectional Encoder Representations from Transformers
GPT: Generative Pre-trained Transformer
LLMs: Large language models
API: Application programming interface
ChatGPT: Chat Generative Pre-trained Transformer
JMLE: Japan Medical Licensing Examination
NBME: National Board of Medical Examiners
USMLE: United States Medical Licensing Examination
